# Obesity changes the human gut mycobiome

**DOI:** 10.1038/srep14600

**Published:** 2015-10-12

**Authors:** M. Mar Rodríguez, Daniel Pérez, Felipe Javier Chaves, Eduardo Esteve, Pablo Marin-Garcia, Gemma Xifra, Joan Vendrell, Mariona Jové, Reinald Pamplona, Wifredo Ricart, Manuel Portero-Otin, Matilde R. Chacón, José Manuel Fernández Real

**Affiliations:** 1 Department of Diabetes, Endocrinology and Nutrition, Institut d’Investigacio´ Biomèdica de Girona (IdIBGi), CIBEROBN (CB06/03/010) and Instituto de Salud Carlos III (ISCIII), Girona, Spain; 2 Genotyping and Genetic Diagnosis Unit, Fundación de Investigación del Hospital Clínico de Valencia-INCLIVA, Valencia, Spain; 3 Hospital Universitari de Tarragona Joan XXIII. IISPV. CIBERDEM. Universitat Rovira i Virgili. Tarragona Spain; 4 Departamento de Medicina Experimental, Universitat de Lleida-IRBLleida. Lleida, Spain

## Abstract

The human intestine is home to a diverse range of bacterial and fungal species, forming an ecological community that contributes to normal physiology and disease susceptibility. Here, the fungal microbiota (mycobiome) in obese and non-obese subjects was characterized using Internal Transcribed Spacer (ITS)-based sequencing. The results demonstrate that obese patients could be discriminated by their specific fungal composition, which also distinguished metabolically “healthy” from “unhealthy” obesity. Clusters according to genus abundance co-segregated with body fatness, fasting triglycerides and HDL-cholesterol. A preliminary link to metabolites such as hexadecanedioic acid, caproic acid and N-acetyl-L-glutamic acid was also found. *Mucor racemosus* and *M. fuscus* were the species more represented in non-obese subjects compared to obese counterparts. Interestingly, the decreased relative abundance of the *Mucor* genus in obese subjects was reversible upon weight loss. Collectively, these findings suggest that manipulation of gut mycobiome communities might be a novel target in the treatment of obesity.

The human gut is a robust ecosystem composed of a dynamic microbial community, collectively termed microbiota, which have important roles in the acquisition of energy from foods and the regulation of host physiology through immune modulation^1^,^2^. The finely tuned equilibrium between the host and microbiota may be disrupted when changes in the latter, described as “dysbiosis”, occur in the context of prevalent disorders such as obesity, diabetes and metabolic inflammation^2^,^3^.

Bacteria are the most abundant components of the human gut microbiome^1^,^4^,^5^. The composition of distal gut microbiota is known to be altered in obesity and obese individuals generally harbour a decreased ratio of Bacteroidetes to Firmicutes^1^,^2^,^4^. The relationship between gut microbiota and the emergence of obesity^4^,^6^,^7^ might be causal, opening a myriad of possibilities for novel treatment approaches.

The mycobiome, referring principally to the fungal component of microbiota, comprises approximately 0.03–2% of total gut microorganisms and is an integral part of the gastrointestinal tract^8^,^9^,^10^,^11^. The diversity of fungi inhabiting the human gut remains poorly explored^9^,^12^,^13^,^14^. A fungal cell is >100-fold larger than a bacterial cell, and thus fungi represent substantially greater biomass than is suggested by the number of available genomes^3^.

Next generation sequencing has been valuable for characterizing the human gut mycobiome^3^,^12^,^15^,^16^. Studies have shown that the human gut is home to more than 66 genera and 184 species of fungi, with *Candida, Saccharomyces* and *Cladosporium* being particularly common^3^,^9^,^11^,^12^,^15^,^17^,^18^. In mice, the majority of gut fungi are indigenous to the intestine since only a few of the most common gut fungi were found in mouse food^16^.

Mycobiome dysbiosis is relevant in inflammatory diseases such Crohn’s disease or ulcerative colitis^16^,^17^,^18^. As yet, no studies have addressed the role of gut fungal microflora in obesity and related diseases.

Here, we used Multitag Pyrosequencing (MTPS) to evaluate fungal diversity in faecal samples from obese and non-obese patients using internal transcribed spacer (ITS) primers, which have broad fungal specificity. We found that the faecal mycobiome is disturbed in obese patients in comparison with non-obese subjects, in parallel with alterations in glucose and lipid metabolism. We observed an optimal grouping of individuals according to genus abundance, that clustered together with metabolic phenotypes. Additionally, we found a dynamic relationship between adiposity and the gut mycobiome after weight loss, indicating that manipulation of gut mycobime communities could be a novel approach in the treatment of obesity.

## Results

### Characteristics of the studied participants

A total of 52 Caucasian subjects were recruited at the Hospital Universitari Dr. Josep Trueta, Girona (Spain) (Supplementary Table 1). The patients were matched by age and sex and stratified according to BMI in order to evaluate the associations between faecal fungi and obesity. Clinical and analytical characteristics according to obesity status are shown in Table 1.

### Taxonomic description and relative abundance of fungi

The Internal Transcribed Spacer (ITS)-based sequencing effort yielded 126704 sequence reads, of which 102891 corresponded to known fungi belonging to 5 different phyla, 13 classes, 50 families and 75 different genera (Supplementary Table 2).

Fungal sequences were detected in every sample analyzed. Less than 1% of sequences corresponding to unknown fungi were detected in 48% of subjects and between 1 and 20% in 17.3% of subjects. The majority of classes, families and genera belonged to the phylum Ascomycota, which corresponds to the largest fungi phylum, and were detected in all samples studied. The phyla Basidiomycota and Zygomycota were detected in 90.38% and 42.30% of samples, respectively. The phyla Chytridiomycota and Neocallimastigomycota, which include a small number of fungi, were present only in two subjects.

At the class level, Saccharomycetes was the most predominant, and was detected in every sample analyzed, followed by Eurotiomycetes and Dothideomycetes, present in 92.3% and 61.53% of the samples, respectively; all belonging to the phylum Ascomycota. The most abundant class of the Basidiomycota phylum was Agaricomycetes, which was present in 46.15% of the samples.

The most abundantly detected families were: Aspergillaceae (86%), belonging to the class Eurotiomycetes, and Dipodascaceae (52%) and Saccharomycetaceae (67.3%) belonging to the class Sacharomycetes. Finally, the family Mucoraceae, one of the three family sequences from the phylum Zygomycota, was identified in 38% of samples.

The most prevalent genera in our analysed samples were *Penicillium* (present in 73% of the samples), followed by *Candida* (55%), *Saccharomyces* (55%), *Mucor* (38%) and *Aspergillus* (35%). The relative proportions of the most abundant genera detected by pyrosequencing in each patient are shown in Supplementary Figure 1.

### Obese mycobiome

**Figure 1:**
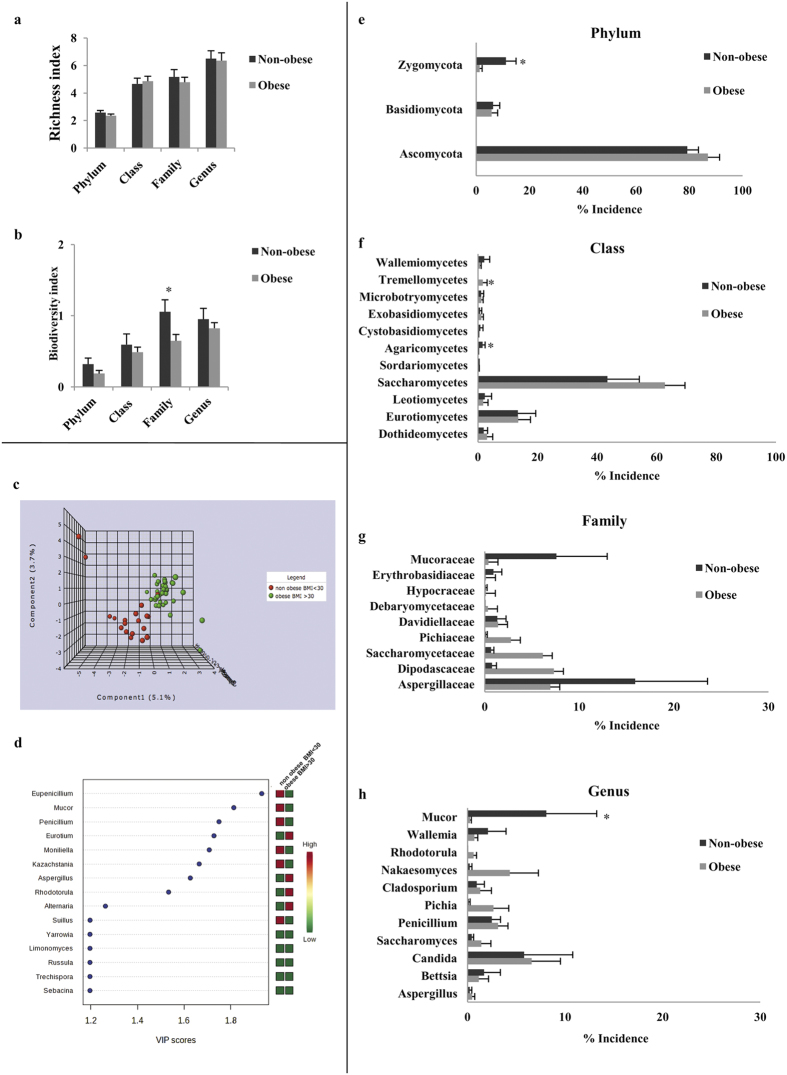
Fungal distribution in obese and non-obese subjects. (**a**) Fungal richness and (**b**) fungal biodiversity observed in obese and non-obese faecal samples. (**c**) Mycobiota genus abundance defined by PLSDA in obese and non-obese individuals. (**d**) Variable importance in projection of the first component of the PLSDA model. Frequencies of detected fungi in each phylum (**e**), class (**f**), family (**g**) and genus (**h**) between obese and non-obese patients. Mean values ± s.e (bars) are plotted, *p < 0.05.

No differences were detected in the richness of the mycobiome between non-obese and obese subjects (Fig. 1a). However, family biodiversity was significantly lower in obese subjects compared with non-obese individuals, and a tendency towards increased biodiversity at other levels was also found in non-obese individuals (Fig. 1b).

Obese and non-obese patients could be distinguished by their specific fungal composition (Fig. 1c,d). The relative abundance of the two major phyla, Ascomycota and Basidiomycota, was not significantly different between obese and non-obese subjects; however, the minor phylum Zygomycota was significantly under-represented among obese subjects (p = 0.031) (Fig. 1e).

At the class level, Tremellomycetes appeared only in a subset of obese subjects, whereas it was completely absent in non-obese subjects (p = 0.028). In contrast, the class Agaricomycetes was significantly more abundant in non-obese patients compared to obese patients (p = 0.017) (Fig. 1f).

At the family level, no significant differences were observed between obese and non-obese subjects. Nonetheless, Aspergillaceae (15.91%) and Mucoraceae (7.58%) were the most prevalent families in non-obese subjects, while Dipodascaceae (7.31%), Aspergillaceae (6.93%) and Saccharomycetaceae (6.13%) were the most abundant in obese subjects (Fig. 1g).

*Candida, Nakaseomyces* and *Penicillium* (present in 6.57%, 4.33% and 3.12%, respectively) were the most abundant genera detected in obese patients (Fig. 1h)., *Mucor* was the most prevalent genus in non-obese patients (8.07%), followed by *Candida* and *Penicillium* (5.79% and 2.49% respectively).

### Sample clusters according to genus abundance: relation with metabolic parameters

We used multivariate analyses to further evaluate the potential differences in the composition of the mycobiome with respect to obesity. A Partial Least Square Discriminant Analysis (PLSDA) revealed that a model with a high accuracy (three component model accuracy 0.77; R^2^ 0.83 and Q^2^ 0.027) could distinguish obese from non-obese mycobiomes (Fig. 1c,d). In order to examine potential overfitting, we also tested as alternative systems for classification random forest-type classifications and recursive support vector machine, by using all genera of the samples. Regarding random forest-type classification, the system produced a relatively low classification error for obese individuals (18%), with high abundance of *Aspergillus* genus and low abundance of *Mucor, Penicillium, Saccharomyces* and *Eupenicillium* genera contributing to obesity (Supplementary Fig. 2). Concerning the recursive support vector machine, this system generated a 10-genus model with an overall error rate near 30% (Supplementary Fig. 3). In this model, low abundance genera, such as *Moniliella, Paralepista, Fuscosporia, Limonomyces* were present in most of the models tested, but also low *Eupenicilium* abundance was present in obese individuals. Further, by including a model of only *Eupenicillium, Mucor, Penicillium, Moniliella* and *Eurotium,* PLSDA model accuracy was 79.3%, with low discovery rate (permutation test, p = 0.04), suggesting that all these genera are fairly associated with obesity (data not shown).

**Figure 2:**
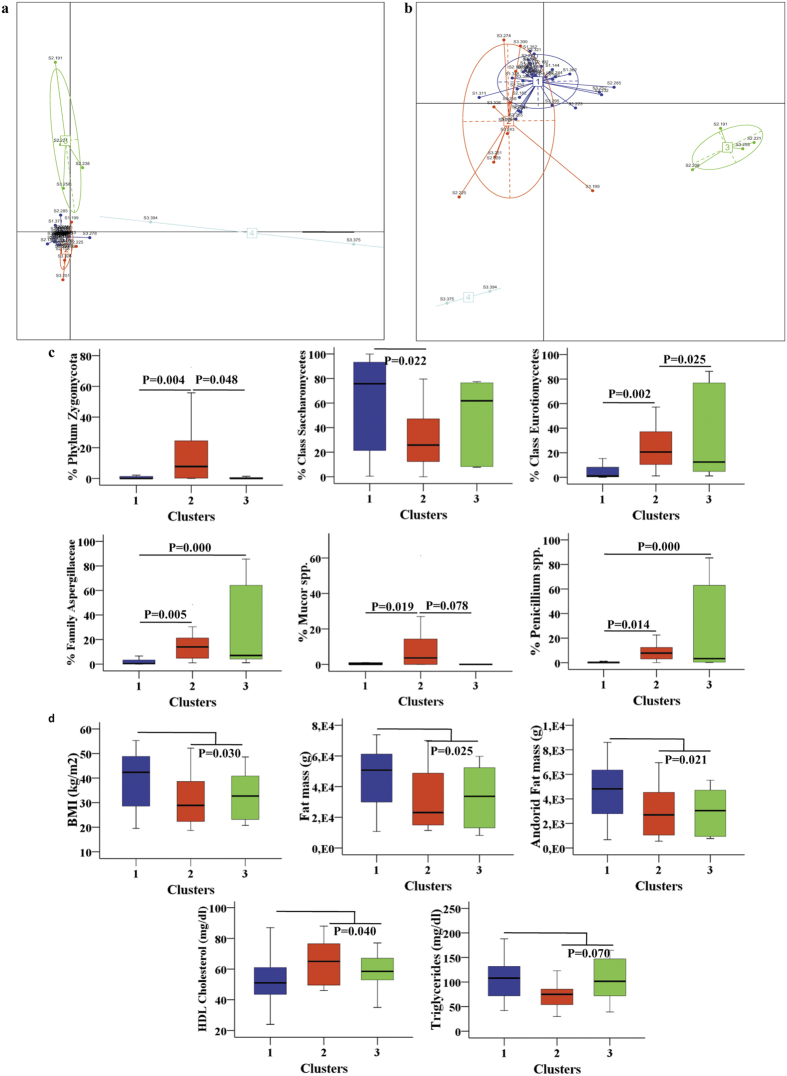
Phylogenetic and metabolic differences between clusters (**a**) Class analyses and (**b**), Cluster of patients based on fungi genus identified by pyrosequencing-based ITS- sequences using Principal Coordinate Analyses (PCA) (**c**), Abundance of the main contributors of each cluster. The coloured scale blox plot, blue, red and green represents cluster 1, 2 and 3 respectively. (**d**) Cluster associations with clinical and anthropometrical data (body fat and lipid profile). BMI: Body mass index, HDL-cholesterol: high-density lipoprotein.

**Figure 3:**
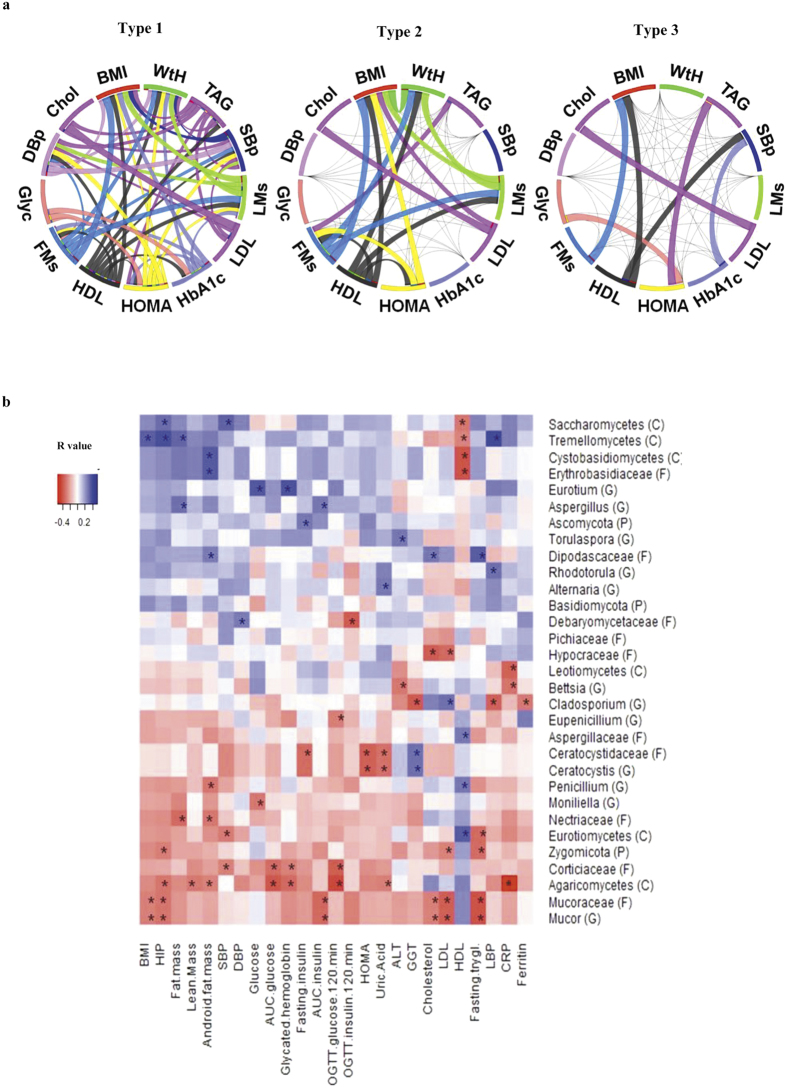
Relationships between human mycobiome and anthropometric and metabolic parameters. (**a**) Circos table viewer plot of significant correlations (Spearman test, at least p < 0.05) among anthropometric, metabolic and cardiovascular parameters in the identified clusters. The different Circos graphs depict that these associations are present in individuals belonging to specific clusters while in others the correlation disappears. Black color indicates negative correlations and edge width of lines connecting segments is proportional to correlation coefficient (**b**) Heat map showing associations of phylum, class, family and genus with clinical and anthropometrical data. The heat map is organized with fungi in rows and metabolic parameters in columns. *p < 0.05. Abbreviations key: P: Phylum, F: Family, C: Class, G: Genus, BMI: Body mass index, SBp: Systolic blood pressure, DBp: Diastolic blood pressure, LMs: Lean mass, FMs: Fat mass, WtH: waist to hip ratio, TAG: triacylglycerides, Glyc: fasting glycemia, Chol: total cholesterol, GTT: Glucose tolerance test, AUC: Area under the curve, HOMA: homeostatic model assessment insulin resistance, AST: Aspartate aminotransferase, ALT: Alanine aminotransferase, GGT: Gamma-glutamyltranferase, LDL-cholesterol: low-density lipoprotein, HDL-cholesterol: high-density lipoprotein, LBP: Lipopolysaccharide-binding protein, CRP: C-reactive protein.

Next, the relative genus abundances were used for optimal grouping (clustering) of individuals according to the *Calinksi-Harabasz* index. Interestingly, these tests uncovered the presence of 3 potential clusters (Fig. 2a,b) that differed according to the relative abundance of *Penicillium* spp., *Mucor* spp., Aspergillaceae, Saccharomycetes, Eurotiomycetes and Zygomycota (Fig. 2c). Strikingly, these clusters were associated with body fatness (BMI, DEXA-measured fat mass and android fat mass), and also with fasting triglycerides and HDL- cholesterol (Fig. 2d). The relationships among anthropometric and metabolic parameters were more marked between subjects harbouring cluster 1 (Fig. 3a).

### Association with anthropometrical and metabolic parameters

Heat map analysis demonstrated significant associations among anthropometric and metabolic parameters and fungal taxa (Fig. 3b). The relative abundance of the phylum Zygomycota, class Agaricomycetes, families Mucoraceae and Nectriaceae and genus *Mucor* and *Penicillium* correlated negatively with parameters of body fatness such as BMI, fat mass, android fat mass and hip circumference. Conversely, the relative abundance of the classes Sacharomycetes, Tremellomycetes, Cystobasidiomycetes, and families Erythrobasidiaceae and Dipodascaceae and genus *Aspergillus* was positively associated with adiposity. Moreover, serum total cholesterol, LDL-cholesterol and fasting triglycerides were negatively linked to the relative abundance of sequences belonging to phylum Zygomycota, and also to the class Eurotiomycetes, families Mucoraceae, Hypocraceae and genus *Mucor*. Conversely, the relative abundance of those sequences belonging to the family Dipodascaceae was positively associated with serum total cholesterol and fasting triglycerides. The relative abundance of fungi belonging to the class Eurotiomycetes, family Aspergillaceae and genus *Penicillium* was positively correlated with HDL-cholesterol, whereas the abundance of the classes Saccharomycetes, Tremellomycetes, Cystobasidiomycetes and family Erythrobasidiaceae was negatively correlated with this parameter (Fig. 3b).

Fascinatingly, the relative abundance of some fungi was linked to parameters of glucose metabolism. Specifically, the relative abundance of fungi of the class Agaricomycetes, and families Mucoraceae, Ceratocystidaceae, Corticiaceae, Debariomycetaceae, genus *Mucor, Monilliela, Ceratocystis* and *Eupenicillium* were negatively associated with HOMA value, glycated haemoglobin and AUC of glucose and insulin during oral glucose tolerance testing. Conversely, the relative abundance of the genus *Eurotium* was positively associated with fasting glucose and glycated haemoglobin, while the phylum Ascomycota was positively associated with fasting insulin, and the genus *Aspergillus* correlated positively with AUC insulin. Of note, we also observed associations with parameters of metabolic inflammation (C-reactive protein and lipopolysaccharide binding protein) (Fig. 3b).

### Associations of the gut mycobiome with “healthy” and “unhealthy” obesity

**Figure 4:**
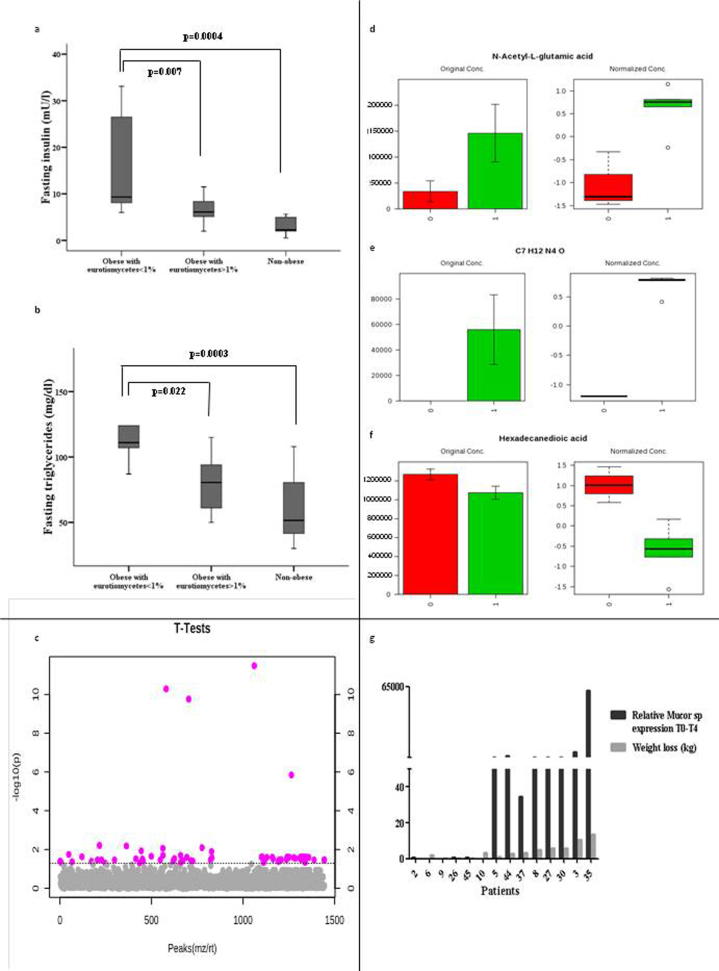
Metabolic associations of the fungal faecal communities within obese subjects and their relationship with body fat. Classification of patients according to Eurotiomycetes abundance in relation to (**a**) fasting insulin (**b**) fasting triglycerides (**c**) Individuals with Eurotiomycetes abundance <1% and those with >1% present significant differences (p < 0.05, Student’s t test) in the plasma concentration of several metabolites, coloured in pink. Profiles panels (both normalized and raw data) from representative metabolites. (**d**) N-acetyl-L-glutamic acid (**e**) Caproic acid (**f**), Hexadecanedioic acid 0: individuals with Eurotiomycetes <1% and 1: individuals with Eurotiomycetes >1%. (**g**) *Mucor* is associated with specific abundance of selected metabolites.*p < 0.050.

Amongst obese individuals, we observed significant associations between the relative abundance of sequences belonging to the Eurotiomycetes class (belonging to Ascomycota phylum) and metabolic parameters (Supplementary Table 3). Interestingly, although the mean relative abundance of Eurotiomycetes was similar between obese (13.02%) and non-obese (13.40%) subjects, 40% of obese patients had an abundance lower than 1%. We observed that obese subjects with less than 1% of sequences belonging to Eurotiomycetes in faecal samples had a more pronounced dyslipidemic profile, increased fasting triglycerides and increased total cholesterol concomitant with increased fasting hyperinsulinemia, compared with obese patients with Eurotiomycetes >1% (p = 0.022, p = 0.036 and p = 0.007, respectively) (Supplementary Table 3) (Fig. 4a,b), and a tendency towards increased HOMA and LDL-cholesterol (p = 0.063 and p = 0.055, respectively) (Supplementary Table 3). Interestingly, no significant differences in fasting insulin and fasting triglycerides were found between obese subjects with Eurotiomycetes present in >1% and non-obese subjects (Fig. 4a,b).

We performed a plasma metabolomics profile in 8 subjects (Supplementary Table 4). Several metabolites differed significantly in concentrations between individuals with Eurotiomycetes abundance <1% and those with >1% (Fig. 4c). Remarkably, the relative abundance of Eurotiomycetes was associated with plasma concentrations of N-acetyl-L-glutamic acid, caproic acid and hexadecanedioic acid, among other metabolites (Fig. 4d–f). The abundance of *Mucor* genus was also found significantly correlated with certain metabolites (Fig. 4g).

### Weight loss results in increased relative abundance of the *Mucor* genus

**Figure 5:**
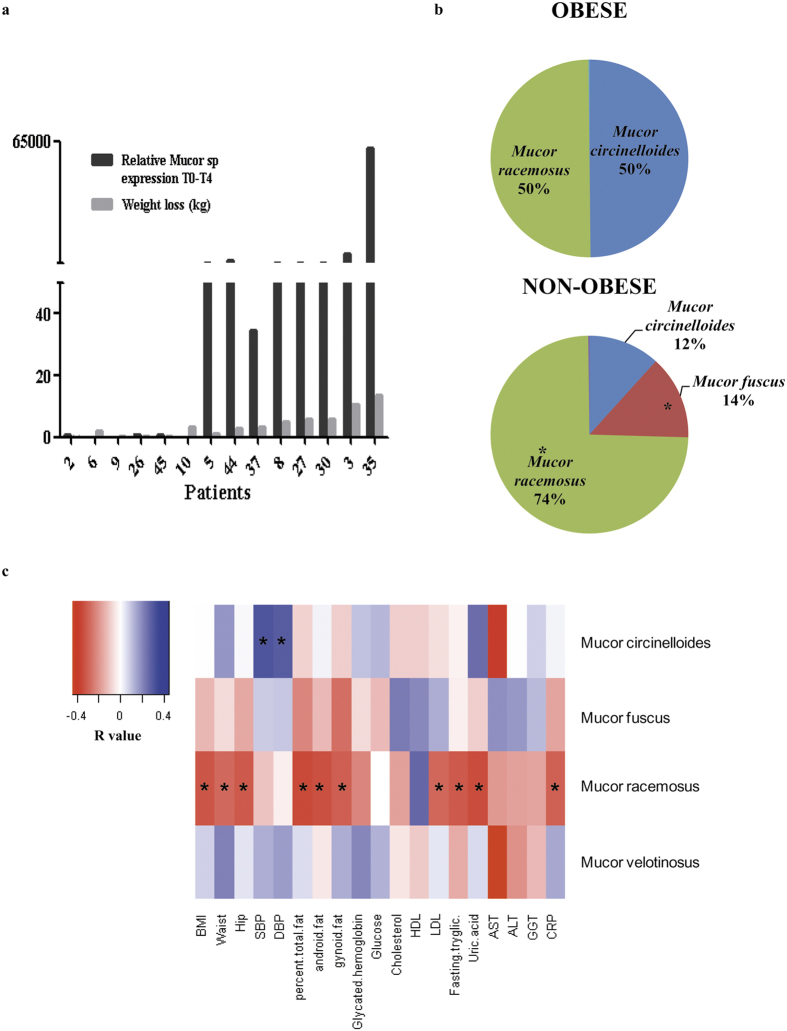
Mucor is associated with obesity. (**a**) Changes in the relative abundance of *Mucor* gene expression and weight loss at time 0 and 4 months after weight loss for every studied patient. (**b**) *Mucor* species distribution in obese and non-obese studied fecal samples. *p < 0.050. (**c**) Heat map showing associations of *Mucor* spp. with clinical and anthropometrical data. The heat map is organized with *Mucor* spp. in rows and metabolic parameters in columns. *p < 0.05. Abbreviations key: BMI: Body mass index, SBP: Systolic blood pressure, DBP: Diastolic blood pressure, HDL-cholesterol: high-density lipoprotein, LDL-cholesterol: low-density lipoprotein, AST: Aspartate aminotransferase, ALT: Alanine aminotransferase, GGT: Gamma-glutamyltranferase, CRP: C-reactive protein.

Since the *Mucor* genus was significantly more abundant in non-obese subjects, we investigated the potential relationship between gut mycobiome ecology and body fat. We evaluated the relative abundance of *Mucor* spp. in 14 patients by real time PCR before and after diet-induced weight loss. Results showed that the relative abundance of *Mucor* spp. for each subject paralleled the degree of weight loss (Fig. 5a). Additionally, pyrosequencing of *Mucor* positive patients showed that *M. fuscus, M. circineloides, M. velotinosus* and *M. racemosus* were the species identified in *Mucor* genus positive faeces. In non-obese patients, *M. racemosus* and *M. fuscus* were significantly more present (p = 0.0099 and p = 0.0316, respectively) when compared with obese patients (Fig. 5b).

The relative abundance of *M. racemosus* was negatively associated with BMI, waist circumference, hip circumference, % total fat, fat android distribution, fat gynoid distribution, LDL-cholesterol, fasting triglycerides, uric acid and CRP (Fig. 5c).

## Discussion

This study shows that the mycobiome of obese subjects has an increased presence of the phylum Ascomycota, class Sacharomycetes and families Dipodascaceae and Saccharomycetaceae and, an increased relative abundance of fungi belonging to class Tremellomycetes, compared with non-obese subjects. Although fungi are known to be linked to a number of gastrointestinal diseases^11^,^14^,^15^,^17^,^18^, the association between fungal microflora and obesity is novel. A clear tendency towards decreased diversity was observed in obese subjects. These findings are consistent with previously reported data on bacterial diversity in the obese state^19^ and are contrary to the observed increase in fungal diversity in patients with inflammatory bowel disease or chronic hepatitis B^13^,^17^,^18^. Overall, these findings indicate that the fungal gut mycobiome seems to be disturbed in obese patients, in association with alterations in lipid and glucose metabolism.

The relative abundance of some fungi was linked to adiposity and related metabolic disorders including insulin resistance, dyslipidaemia, blood pressure and inflammatory activity. For example, the phylum Ascomycota, classes Sacharomycetes, Tremellomycetes and Cystobasidiomycetes, families Erythrobasidiaceae and Dipodascaceae and genera *Aspergillus, Eurotium* and *Rhodotorula*, all increased with the occurrence of metabolic abnormalities. Conversely, the relative abundance of fungi belonging to the phylum Zygomycota, classes Agaricomycetes and Eurotiomycetes, families Mucoraceae, Nectriaceae, Ceratocystidaceae, Corticiaceae, Debariomycetaceae and Hypocraceae, and genera *Mucor, Penicillium, Monilliela* and *Ceratocystis* were associated with protection from these metabolic disorders. The finding that systemic inflammation is associated with obesity suggests that the obesity-related bacterial gut composition has a proinflammatory effect^19^. This is similar to our observations with Tremellomycetes. This class was not only significantly more abundant in obese subjects, but also correlated positively with inflammatory parameters. Of note, some metabolites from the *Penicillium* genus have been shown to exhibit anti-inflammatory and insulin sensitizing activities^20^.

Interestingly, the presence of some fungal communities was associated with metabolically healthy obese subjects. Although the relative abundance of Eurotiomycetes was similar in obese and non-obese subjects, 40% of obese subjects had an abundance less than 1%, and this was associated with a worse metabolic glucose and lipid profile. Moreover, those obese subjects with >1% Eurotiomycetes had similar fasting insulin and fasting triglycerides compared with non-obese subjects.

N-acetyl-L-glutamic acid is known to be metabolized by some members of the Ascomycota phylum, to which Eurotiomycetes belongs^21^. Interestingly, N-acetyl-L-glutamic acid salts have been shown to exhibit natriuretic effects, resulting in the lowering of blood pressure^22^. Furthermore, hexadecanedioic acid, associated with a decreased abundance of Eurotiomycetes, has been demonstrated to exhibit antimycotic activity^23^. Further studies should be performed to experimentally address whether the metabolites described here correlate only with the expansion (or decrease) of specific fungi, or whether they have a direct effect on these fungi.

*Candida* spp. have been successfully identified from the intestine of healthy individuals^9^,^17^. Some studies have suggested a link between expansion in *Candida* spp. and diabetes^24^ and inflammatory disorders of the gastrointestinal tract^17^,^25^, with a possible active role for *Candida albicans*^26^. In this sense, we have not observed differences of the relative expression of *Candida albicans* in obesity (data not shown).

In our study, the *Mucor* genus was the most prevalent genus in non-obese subjects; specifically, *M. racemosus* and *M. fuscus* were the more representative species in these subjects. *Mucor* sp. has a cell wall polysaccharide composition based on chitin-chitosan^27^, and multi-functional roles of this polysaccharide as a protective agent against obesity have been described^28^. *M racemosus* has been described as source of chitosan^29^. Future animal studies will help to evaluate the role of the identified *Mucor* spp. in obesity.

Mechanistically, we also demonstrate that the relative abundance of the *Mucor* genus increased after weight loss in obese subjects in a manner analogous to Bacteroidetes, which increases also after weight loss^4^,^30^. However, a larger study should be carried out to evaluate the significance of these findings.

At the bacterial level, three predominant enterotypes have been identified based on variations in the levels of three different genera (*Bacteroides, Prevotella* and *Ruminococcus)*^10^,^31^. Here we identified 3 different clusters; strikingly, cluster 1 was significantly associated with adiposity markers and dyslipidemia, hinting at a possible target for treatment. We observed that the major shift in fungal populations associated with cluster 3 mirror similar dramatic changes in the bacterial communities (ratio Firmicutes/Bateriodetes) than cluster 1 and 2 (data not shown). One could argue that 4 samples that fall in the less abundant cluster 3 are actually outliers. In order to exclude that proposed clusters were not derived from outlying measures, we performed Grubb’s outlier test on the matrix of Jensen-Shannon distances, by employing the R package “outliers”, and found no significant outliers. However the number of subjects studied within this cluster was too low to reach to any conclusion. We acknowledge that further cluster validation is required for a generalization of these results as potential “enteromycotypes”.

Finally, fungal-fungal interaction analysis showed that, in obese patients, the Ascomycota phylum negatively correlated with Basidiomycota and Zygomycota phyla. Noteworthy, among non-obese subjects, a significant negative association was observed between Pichiaceae and Dipodascaceae. The same antagonistic relationship between *Candida* (Family Dipodascaceae) and *Pichia* (Family Pichiaceae) has been observed in oral mycobiota of HIV-infected patients^32^.

In summary, this study represents the first analysis of the human mycobiome in obesity and associated metabolic disorders. There is limited knowledge on gut bacterial-fungal interactions and their role in health and disease. An understanding of cross-kingdom microbial interactions within the context of health and disease holds considerable promise to facilitate the discovery of potential preventative and therapeutic targets. Further analysis on the metabolic road-maps involving fungal and bacterial genomes will be needed to unequivocally unravel new avenues in inter-kingdom metabolic dependencies.

## Methods

### Subjects and Sample Collection

Two cohorts were used to perform the study. In cohort 1, 52 subjects were recruited at Hospital Universitari Dr. Josep Trueta, Girona (Spain) (Supplementary Table 1). All subjects were Caucasian and reported that their body weight had been stable for at least 3 months before the study. These subjects had no systemic disease other than obesity or dyslipidaemia, were free of any infection in the month before the study and did not undergo treatment with drugs that affect glucose metabolism or antibiotics. Liver and renal diseases were specifically excluded by biochemical work-up.

Cohort 2 included 14 overweight and obese patients from the Hospital Universitari Dr. Josep Trueta. The subjects were 7 men and 7 women, aged 54.2 ± 8.7 years, with a mean BMI of 33.4 ± 7.4 kg/m^2^. Subjects were instructed to maintain a 20 kcal/kg balanced diet. Measurements and samples were taken at baseline and 16 weeks after starting the diet. At the end of this period, mean BMI was 31.5 ± 7.3 kg/m^2^ (p = 0.005 vs. baseline).

All experiments were performed in accordance with approved guidelines and regulations. All experimental protocols were approved by the Ethics Committee of the Hospital Universitari Dr. Josep Trueta (*Comitè d’Ètica d’Investigació Clínica*, CEIC, ethical approval number 2009046). Informed consent was obtained from all participants.

### Clinical and biochemical variables

Each patient underwent evaluation of anthropometric and laboratory parameters on the same day. Blood pressure was measured using a blood pressure monitor (Hem-703C, Omron, Barcelona, Spain), with the subject seated and after 5 minutes of rest. Three readings were obtained and the mean value was used in the analyses.

### Body composition

Fat mass was evaluated by dual-energy x-ray absorptiometry (DEXA) using a GE Lunar Prodigy Oracle densitometer (GE Healthcare, enCore software version 13.2). Whole body composition (fat mass and fat-free soft tissue mass) was obtained by trained personnel according to standard procedures. Obesity was considered as a BMI ≥30 kg/m^2^. Android fat mass was calculated using regions of interest at the abdominal level in DEXA scans.

### Analytical methods

After 8 h of fasting, blood was drawn for the measurement of fasting plasma lipids, glucose and insulin. Serum glucose concentrations were measured in duplicate by the glucose oxidase method using a Beckman Glucose Analyser II (Beckman Instruments, Brea, CA). Intra-assay and inter-assay coefficients of variation (CV) were less than 4%. Plasma lipid determinations were performed on a Roche/Hitachi Cobas c 711 autoanalyzer (Roche Diagnostics GmbH, Mannheim, Germany). Total serum cholesterol was measured by an enzymatic colorimetric cholesterol esterase/cholesterol oxidase/peroxidase reaction (Cobas CHOL2). HDL cholesterol was quantified by a homogeneous enzymatic colorimetric esterase/cholesterol oxidase/peroxidase reaction (Cobas HDLC3). LDL cholesterol was calculated using the Friedewald formula. Total serum triglycerides were measured by an enzymatic colorimetric glycerol phosphate oxidase and peroxidase reaction (Cobas TRIGL).

Glycated haemoglobin (HbA1c) was measured by high-pressure liquid chromatography using a fully automated glycosylated haemoglobin analyzer system (Hitachi L-9100). Serum insulin was measured in duplicate using a monoclonal immunoradiometric assay (Medgenix Diagnostics, Fleunes, Belgium). The intra-assay CV was 5.2% at a concentration of 10 mU/l and 3.4% at 130 mU/l. The inter-assay CVs were 6.9 and 4.5% at 14 and 89 mU/l, respectively. The homeostatic model assessment insulin resistance index (HOMA-IR) was calculated by the formula: (serum glucose (mmol/l) x serum insulin (mU/l))/22.5. A standard 75 g oral glucose tolerance test was performed after an overnight fast and venous blood samples were drawn at time points 0 30, 60, 90 and 120 min for determination of plasma glucose and insulin. Area under the curve of glucose (AUC-glucose) and insulin (AUC-insulin) concentrations during the 120 min of the 75 g oral glucose tolerance test was then calculated using the trapezoid method. C-reactive protein (ultrasensitive assay; Beckman, Fullerton, CA) was determined by a routine laboratory test, with intra- and interassay CVs of 4%. The lower limit of detection is 0.02 mg/l. Serum ferritin was measured by microparticle enzyme immunoassay (AxSYM™; Abbot Laboratories) with intra- and interassay CVs < 6%. Serum lipopolysaccharide binding protein (LBP) levels were measured with an ELISA kit (HK315-02, HyCult Biotech Inc., Uden, The Netherlands). Serum samples were diluted and assayed according to the manufacturer’s instructions. Intra- and inter-assay CVs for all the determinations were between 5 and 10%. Serum alanine aminotransferase (ALT), aspartate aminotransferase (AST) and gamma-glutamyltransferase (GGT) levels were determined using enzymatic methods. Uric acid was determined by routine laboratory tests.

### Stool processing and DNA extraction

Stool specimens were obtained from patients using sterile containers and were immediately frozen in liquid nitrogen and stored at −80 °C until analysis. Samples were processed individually using the Fast DNA Spin Kit for faeces (MP Biomedicals, Solon, OH). Briefly, a frozen aliquot (400mg) of each sample was added to a 2 ml tube containing 825 μl sodium phosphate buffer, 275 μl of pre-lysis solution and Lysing matrix E, a mixture of ceramic and silica particles designed to efficiently lyse all stool microorganisms. Each extraction tube was agitated twice for 40 seconds using a Fast Prep FP120 instrument at a speed setting of 6 to ensure proper extraction of fungal DNA, a crucial point in the methodology. Tubes were cooled on ice between the different agitation procedures. DNA extraction was then carried out following the manufacturer’s instructions. The quantity and quality of isolated DNA was determined with a Nanodrop ND-1000 spectrophotometer (Nanodrop Technologies, Wilmington, DE, USA).

### Pyrosequencing analysis

Fungal genomic sequences present in faecal samples were identified by amplification of the internal transcribed spacer-based region (ITS). The ITS primers used span the region between the 3′ end of the 18S gene, including the entire 5.8S gene, and end of the 5′ region of 28S gene. PCR reactions were performed in 15 μl reaction volumes containing 0.33 μM of primer. Two different PCR reactions were performed per sample, using two different pair of primers in a 384-well plate. Both sets of primers contained a universal tag sequence in the second PCR step (5′ = AGGTCAGGATCAACGCTCAAG, 3′ =  CATCTTGCATGATCCAACCTTC). Primer set A; **H1SeqF** GTCATTTAGAGGAAGTAAAAGTCGTAACAAGG and **H1SeqRb** GCT*RY*GTTCTTCATCG*D*TGC. Primer set B; H2**SeqFb** GCA TCG ATG AAG AAC*RY*A GC and primerH2**SeqRb** TTC TTT TCC TCC GCT TAT TGA TAT GC. Standard PCR cycling was performed on a Veriti 384-well Thermal Cycler (Applied Biosystems) with an initial step at 95° for 15 min followed by 35 cycles of 95° for 20 sec, 62° for 30 sec and 72° for 60 sec, and a final step of 72° for 10 min. PCR products were visualized using a Qiaxcel system (Qiagen).

PCR products from the first step were diluted 1/5 with water and were used as templates in the second PCR step in order to tag every PCR product with a sample specific tag multiplex identifier (MID) consisting of a 10 base pair specific sequence. Two μl of the diluted PCR product were used for the second PCR reaction, containing 0.3 μM of each MID. Standard PCR cycling was performed as before. PCR products were visualized using a Qiaxcel system, purified using size-exclusion AMPure SPRI DNA-binding paramagnetic beads (Agencourt Bioscience Corp. Beckman Coulter) and quantified in 96 well-format with the QuantiFluor-ST Fluorometer (Promega) using a PicoGreen® assay (InvitroGen). Samples were then pooled at an approximate equimolar concentration. After pooling, the library was further purified with the Pippin Prep size-exclusion system (SageScience), quantified in 96-well format with the QuantiFluor-ST Fluorometer (Promega) and diluted to 5 × 10^5^ molecules/μl. Five μl of the pools were then subjected to emulsion PCR (emPCR) using the 454 Junior Titanium Series LibA emPCR kit (Roche diagnostics) to perform last amplification step.

### Data Analysis

The 454 multitag pyrosequencing (MTPS) data was de-multiplexed by sorting the sequences into bins based on the barcodes in the samples. A quality control was carried out on the reads for each sample using FastQC (v. 0.10.1) to assess potential problems in the data. TagCleaner (v. 0.12) was used to remove the additional tag sequences (sequencing primers and adaptors) that appeared on the raw reads, which could contain deletions or insertions due to sequencing limitations. This step was followed by the trimming of low quality read ends (both 5′ and 3′) with PRINSEQ (v. 0.19.5), removal of short reads, removal of reads with low mean quality and removal of low complexity reads. The cleaned pyrosequencing data was BLASTed against the NCBI nucleotide collection (nr/nt) database using BLAST from the standalone BLAST+ package. We found that 18.8% of the data did not match to fungal sequences. Fungal reads were assigned taxonomically with MEGAN (v. 5.2.3). This step was optimized to maximize the number of reads (Min Support  =  1, Min Score  =  50.0, Max Expected  =  0.01, Top Percent  =  5.0, Min Complexity  =  0.44). The results were summarized at a variety of taxonomic ranks according to the NCBI taxonomy using the Lowest Common Ancestor (LCA) algorithm in MEGAN. The LCA algorithm assigns taxa to the lowest possible taxonomic rank that presumably reflects the level of sequence variation present in the query sequence compared with reference sequences.

MEGAN classifies each read depending on 4 different types of scenarios: 1) Those reads with a clear species or genera classification get assigned to this taxon. 2) Those with an ambiguous classification (reads match with several different taxa): we report the upper common level of the main matches. 3) Reads in all fungal classes: classified as unknown fungi 4) Those with no matches to fungal sequences: removed from the data analysis but taken into account in the quality control of sequences.

Although parameters were optimized to reach species level as much as possible, when BLAST results classified the sequence as one specific species in most of the hits, MEGAN stops at the higher taxon. We are aware that MEGAN is more conservative than other curated databases, however it is robust enough to be valid for this project and as we are aware of its limitations. Therefore, we decided to provide sequences only up to genus level.

Richness of fungal community was calculated as the number of different taxa for each patient. Biodiversity index was assessed using the Shannon-Weaver index since this is quite insensitive to sample size^18^. This index was calculated with R-bioconductor package vegan (v. 2.0–10).

### Samples clusters according to genus abundance

Samples were clustered based on relative genus abundances using Jensen-Shannon distance and the Partitioning Around Medoids clustering algorithm, previously used for enterotype determination^10^. The results were assessed for the optimal number of clusters using the *Calinski-Harabasz (CH) Index*^33^. These studies, as well as those between class and principal coordinate analyses, were performed by using the r scripts present in http://enterotype.embl.de/enterotypes.html.

### Relative quantification of *Mucor* species by Real-time PCR

*Mucor* species quantification was performed on a 7900HT Fast Real-Time PCR System using the primers and probes described previously^34^, with slight modifications. Mucor (F) 5′ GTC TTT GAA CGC AAC TTG CG 3′, Mucor (R) 5′ CCG CCT GAT TTC AGA TCA AAT T 3′ and Mucor probe: 5′ TTCCAATGAGCACGCCTGTT-MGBNFQ 3′. DNA from *Mucor circinelloides* (CBS277.49), belonging to the mold collection of the Fungal Biodiversity Center (CBS) was used as a positive control. For *Mucor* detection, PCR was performed in a final volume of 20 μl containing 0.5 μM of each primer of the Mucormycetes species, 0.25 μM of the internal control primers and 0.2 μM of *Mucor* spp. probe. For 18S (reference gene) amplification, we used primers, probes and conditions described previously^35^. PCR conditions for *Mucor* spp., 18S and internal control were: 2 min at 50 °C for Uracil-DNA Glycosylase treatment, 10 min at 95 °C for Taq activation, 15 s at 95 °C for denaturation and 1 min at 61.2 °C for annealing and extension (50 cycles). SDS software 2.3 and RQ Manager 1.2 (Applied Biosystems) were used to analyze the results with the comparative threshold cycle (Ct) method (2^−∆∆C^t). C_t_ values for each sample were normalized with the 18S reference gene. All data were expressed as an n-fold difference relative to a calibrator (a mix of DNA from 4 human faecal samples).

### Identification of *Mucor* species by pyrosequencing

For each sample sequenced we created a custom DNA sequencing library specific for *Mucor* sequences. We performed high-throughput shotgun DNA sequencing using the Illumina MiSeq platform 2 × 300 bp. The data analysis consists of two major stages, the denoising and chimera detection stage and the microbial diversity analysis stage. During the denoising and chimera detection stage, denoising is performed using various techniques to remove short sequences, singleton sequences, and noisy reads.

With the erroneous reads removed, chimera detection is performed to aid in the removal of chimeric sequences. Lastly, remaining sequences are then corrected base by base to help remove noise from within each sequence. For this procedure, we used the USEARCH global alignment algorithm and the UCHIME chimera detection software. During the diversity analysis stage, for each sample we determine the taxonomic information for each constituent read. For this procedure we used USEARCH global alignment and UPARSE algorithms and the RDP classifier against a database of high quality sequences derived from the NCBI database. The OTU information was obtained by using MUSCLE aligner and FastTree software.

### Metabolomic analysis

Metabolites were extracted from plasma samples of 8 random samples from cohort 1 with methanol according to a previously described method^36^. Briefly, 30 μl of cold methanol was added to 90 μl of plasma, incubated 1 h at −20 °C and centrifuged for 3 min at 12000 g. The supernatants were recovered, evaporated using a Speed Vac (Thermo Fisher Scientific, Barcelona, Spain) and resuspended in water. We used an ultra-high pressure liquid chromatography (Agilent 1290 LC) system coupled to an electrospray-ionization quadrupole time of flight mass spectrometer (Q-TOF) 6520 instrument (Agilent Technologies, Barcelona, Spain). A column with 1.8 micron particle size was employed, and we performed identification of metabolites using the PCDL database from Agilent (Agilent Technologies, Barcelona, Spain), which uses retention times in a standardized chromatographic system as an orthogonal searchable parameter to complement accurate mass data, according to previously published works^37^.

### Statistical analysis

Statistical analysis was performed using version 19 of the Statistical Package for the Social Sciences (SPSS, Chicago, IL). For clinical and anthropometrical variables, data are expressed as mean  ± SD. Before statistical analysis, normal distribution and homogeneity of the variances was evaluated using Levene’s test, and then variables were log-transformed when necessary. Differences between non-obese and obese subjects were tested with the Mann-Whitney *U* test for non-normally distributed data. For comparison among clusters and fungi, we used the Mann-Whitney *U* test, with Bonferroni corrections for multiple comparisons. Differences between cluster 1 and cluster 2 plus 3, in relation to metabolic parameters, were tested using unpaired *t*-test. Spearman’s correlation coefficient was used to analyze the association between fungi and clinical or metabolic parameters. Partial least square discriminant-analyses were performed using the Metaboanalyst v 3.0 platform^37^. Two-way ANOVA followed by Bonferroni *post hoc* comparison test (Prism 5 software; Graph- Pad Software, Inc., San Diego, CA) was used to assess differences in metabolic parameters according to *Eurotiomycetes* class relative abundance. *P* < 0.05 was considered significant.

## Additional Information

**How to cite this article**: Mar Rodríguez, M. *et al.* Obesity changes the human gut mycobiome. *Sci. Rep.* **5**, 14600; doi: 10.1038/srep14600 (2015).

## Supplementary Material

Supplementary Information

## Figures and Tables

**Table 1:** Clinical and anthropometrical characteristics of the study groups.

Women	Obese subjects	Non-obese subjects	*P*-value
N	27	12	
Age	47.85 ± 7.74	43.08 ± 9.11	0.1
BMI (kg/cm^2^)	45.36 ± 5.52	22.66 ± 3.34	<0.001
Waist (cm)	120.88 ± 11.25	76.91 ± 9.07	<0.001
Hip (cm)	134.81 ± 11.56	100.41 ± 8.74	<0.001
Fat Mass (kg)	57.159 ± 9.328	19.293 ± 7.984	<0.001
Lean Mass (kg)	54.539 ± 6.887	37.892 ± 4.082	<0.001
Android fat mass (kg)	5.562 ± 1.231	1.428 ± 0.841	<0.001
SBP (mmHg)	138.14 ± 18.81	121.83 ± 15.75	0.013
DBP (mmHg)	74.55 ± 10.43	64.83 ± 10.24	0.01
Fasting glucose (mg/dl)	93.77 ± 15.26	89.08 ± 7.84	0.322
Glucose post-OGTT(mg/dl)	134.11 ± 40.15	102.75 ± 29.13	0.02
AUC glucose (mmol/l/60 min)	17680 ± 3838.15	13796.25 ± 2918.35	0.003
Glycated haemoglobin (%)	5.84 ± 0.37	5.44 ± 0.22	0.002
Fasting insulin (mU/l)	11.24 ± 9.18	3.90 ± 3.39	0.001
Insulin post-OGTT(mU/l)	71.96 ± 58.94	32.50 ± 37.08	0.040
AUC insulin (mmol/l/60 minutes)	7075 ± 4189	4094.50 ± 2909.05	0.032
HOMA	2.83 ± 2.82	0.9 ± 0.75	0.003
Uric Acid (mg/dl)	5.20 ± 1.34	3.42 ± 0.77	0.245
AST (U/l)	21.33 ± 12.64	21.5 ± 7.77	0.987
ALT (U/l)	23,66 ± 18,28	19.08 ± 7.08	<0.001
GGT (U/l)	22.22 ± 8.41	18.58 ± 9.86	0.409
Cholesterol (mg/dl)	201.27 ± 37.84	200.83 ± 37.07	0.973
LDL-Cholesterol (mg/dl)	130.51 ± 32.66	115.5 ± 33.33	0.120
HDL-Cholesterol (mg/dl)	48.85 ± 9.54	72.83 ± 16.68	<0.001
Triglycerides (mg/dl)	109.48 ± 43.30	61.5 ± 26.82	<0.001
LBP (μg/ml)	24.99 ± 9.47	16.05 ± 3.03	<0.001
CRP (mg/dl)	0.91 ± 0.47	0.16 ± 0.28	<0.001
Ferritin (ng/ml)	83.4 ± 69.08	54.58 ± 54.40	0.180

Data are given as mean ± SD. BMI: Body mass index, SBP: Systolic Blood Pressure, DBP: Diastolic Blood pressure; OGTT: Oral Glucose Tolerance Test; AUC: Area Under the Curve, HOMA: homeostatic model assessment insulin resistance, AST: Aspartate aminotransferase, ALT: Alanine aminotransferase, GGT: Gamma-glutamyltranferase, LDL-cholesterol: low-density lipoprotein, HDL-cholesterol: high-density lipoprotein; LBP: Lipopolysaccharide-binding protein, CRP: C-reactive protein.
