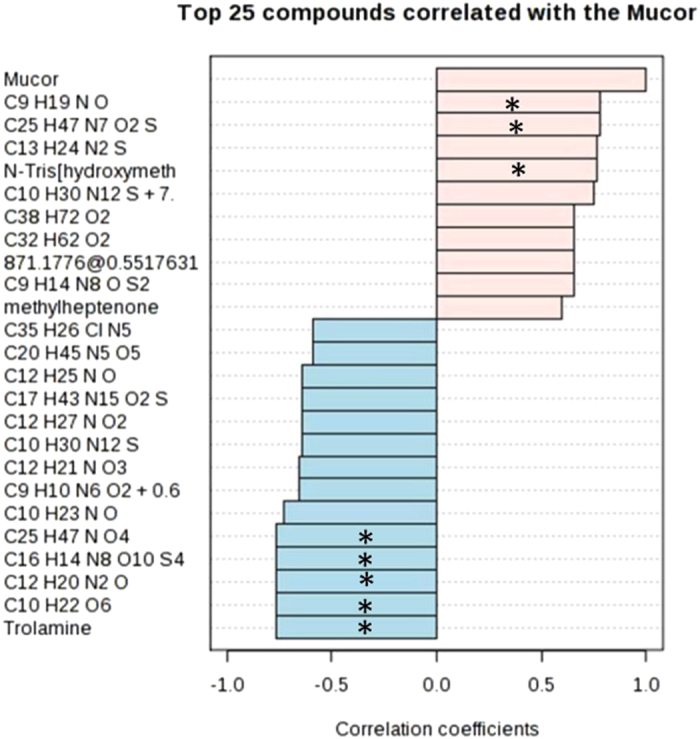# Erratum: Obesity changes the human gut mycobiome

**DOI:** 10.1038/srep21679

**Published:** 2016-02-24

**Authors:** M. Mar Rodríguez, Daniel Pérez, Felipe Javier Chaves, Eduardo Esteve, Pablo Marin-Garcia, Gemma Xifra, Joan Vendrell, Mariona Jové, Reinald Pamplona, Wifredo Ricart, Manuel Portero-Otin, Matilde R. Chacón, José Manuel Fernández Real

Scientific Reports 5: Article number: 1460010.1038/srep14600; published online: 10122015; updated: 02242016.

In this Article, Figure 4g is a duplication of Figure 5a. The correct Figure 4g appears below as Fig. 1.

## Figures and Tables

**Figure 1:**