# Supplementary material for: Obesity changes the human gut mycobiome
Source: Sci Rep. 2015 Oct 12;5:14600. doi: 10.1038/srep14600 (PMC4600977; doi:10.1038/srep14600)
Supplement: Supplementary Information [file srep14600-s1.pdf]

Obesity changes the human gut mycobiome

M. Mar Rodríguez PhD[1], Felipe Javier Chaves PhD[2], Daniel Pérez[2], Eduardo Esteve M.D, PhD[1], Pablo Marin-Garcia PhD[2], Gemma Xifra,[1] Joan Vendrell M.D PhD[3], Mariona Jové[4], Reinald Pamplona M.D, PhD[4], Wifredo Ricart M.D, PhD[1], Manuel Portero-Otin M.D, PhD[4], Matilde R. Chacón PhD[3]*and José Manuel Fernández Real M.D, PhD[1]*

Supplementary figure 1.

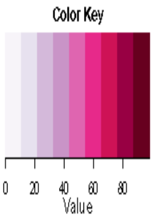

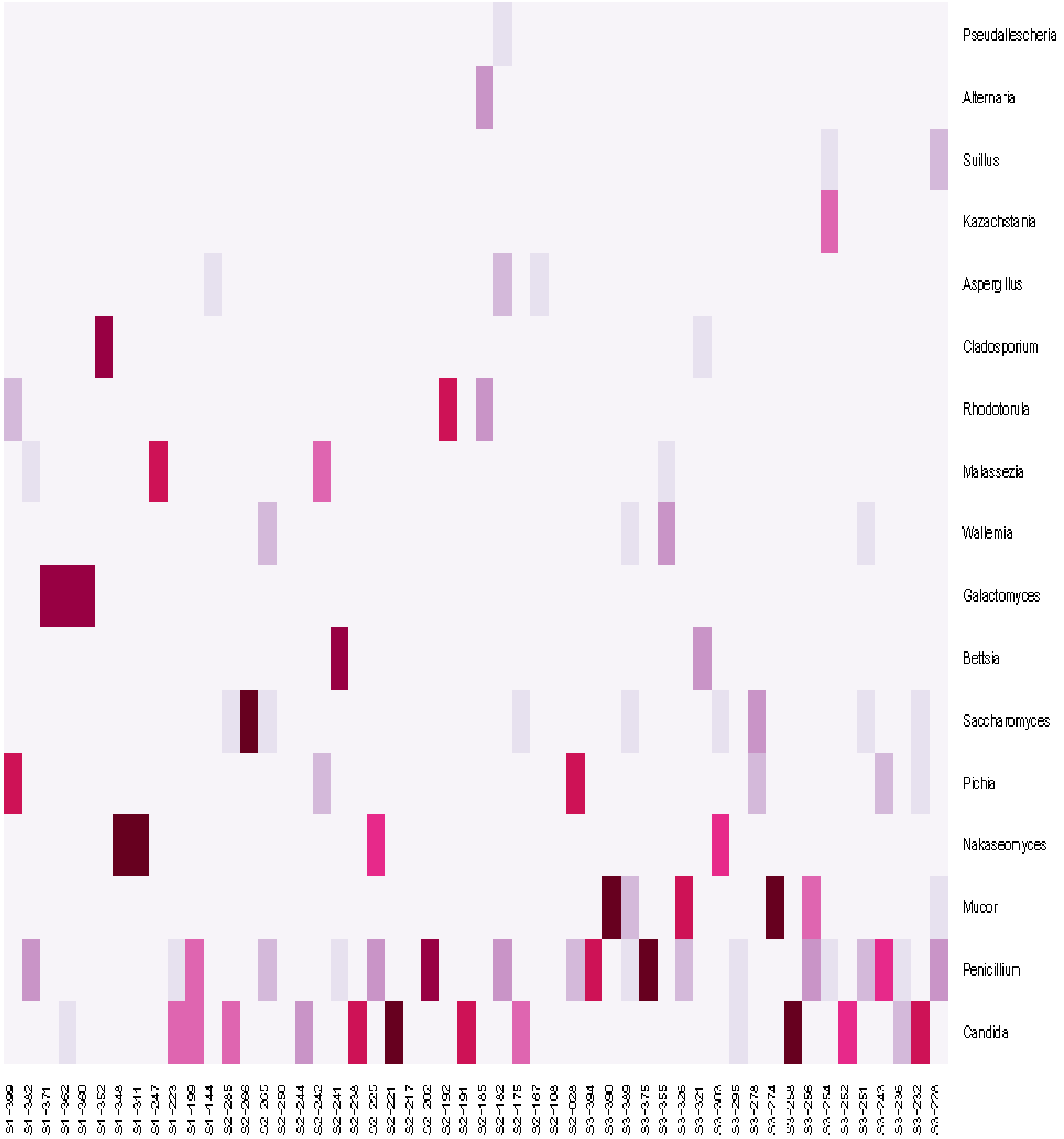

Supplementary table 1| Clinical and anthropometrical data study participants.

	**Subjects**
**N**	52
**Age (years)**	45.25±79.81
**Sex**	43 Women/9 Men
**BMI kg/cm2**	36.85±11.71
**Waist (cm)**	107.13±25.07
**Hip (cm)**	122.09±19.41
**Fat mass (kg)**	42.210±20.059
**Lean Mass (kg)**	50.789±11.429
**Android Fat mass (kg)**	4.063±2.364
**SBP (mmHg)**	131.67±19.91
**DBP (mmHg)**	72.36±11.07
**Fasting glucose (mg/dl)**	90.88±12.53
**Glucose post-OGTT (mg/dl)**	120.4±37.29
**AUC glucose (mmol/l/60min)**	16167.3±3855.16
**Glycated hemoglobin (%)**	5.65±0.37
**Fasting insulin (mU/l)**	9.40±8.78
**Insulin post-OGTT (mg/dl)**	59.48±54.52
**AUC insulin (mmol/l/60 minutes)**	6479.26±4852.27
**HOMA**	2.28±2.46
**Uric Acid (mg/dl)**	4.93±1.58
**AST (U/l)**	23.46±9.41
**ALT (U/l)**	22.19±14.72
**GGT(U/l)**	21.28±10.11
**Cholesterol (mg/dl)**	198.44±37.78
**LDL cholesterol (mg/dl)**	122.61±33.34
**HDL cholesterol (mg/dl)**	55.78±16.08
**Fasting tryglicerides (mg/dl)**	99.86±47.07
**LBP (ug/ml)**	21.39±8.73
**CRP (mg/dl)**	0.59±0.53
**Ferritin (ng/ml)**	105.34±123.50

Data are given as mean±SD. BMI: Body mass index, SBP: Systolic Blood Pressure, DBP: Diastolic Blood pressure, OGTT: Oral Glucose Tolerance Test, AUC: Area Under the Curve, HOMA: homeostatic model assessment insulin resistance, AST: Aspartate aminotransferase, ALT: Alanine aminotransferase, GGT: Gamma-glutamyltranferase, LDL-cholesterol: low-density lipoprotein, HDL-cholesterol: high-density lipoprotein; LBP: Lipopolysaccharide-binding protein, CRP: C-reactive protein.

Suplemmentary Table 2| Taxonomic classification of all fungi sequences identified by pyrosequencing in human faeces.

Phylum	Class	Family	Genus
**Ascomycota**	**Dothideomycetes**	**Pleosporaceae**	*Alternaria*
		**Davidiellaceae**	*Cladosporium*
		**Teratosphaeriaceae**	*Capnobotryella*
		**Dothioraceae**	*Pseudoseptoria*
		**Didymellaceae**	*Phoma*
		**Phaesphaeriaceae**	*Eudarluca*
	**Eurotiomycetes**	**Aspergillaceae**	*Aspergillus*
			Xeromyces
			Eurotium
			Eupenicillium
			Penicillium
		**Trichomaceae**	*Talaromyces*
		**Eremascaceae**	*Eremascus*
		**Herpotrichiellaceae**	*Exophiala*
		**Ascosphaeraceae**	*Ascosphaera*
	**Leotiomycetes**	**incertae sedis**	*Bettsia*
		**Sclerotiniaceae**	
	**Sordariomycetes**	**Hypocraceae**	
		**Plectosphaerellaceae**	*Plectosphaerella*
			Verticillium
		**Ophistomotaceae**	*Phialocephala*
		**Chaetomiaceae**	*Chaetomium*
		**Microascaceae**	*Pseudallescheria*
		**Ceratocystidae**	*Ceratocystis*
		**Gnomiaceae**	
		**Nectriaceae**	*Nectria*
			Fusarium
	**Saccharomycetes**	**Dipodascaceae**	*Candida*
			Dipodascus
			Galactomyces
			Geotrichum
			Yarrowia
		**Pichiaceae**	*Pichia*
		**Debaromycetaceae**	*Debaromyces*
		**Saccharomycetaceae**	*Saccharomyces*
			Nakaseomyces
			Torulaspora
			Eremothecium
			Kazachstania
			Zygosaccharomyces
		**Metschnikowiaceae**	*Metschnikowia*
			Clavispora
		**Sacharomycodaceae**	*Hanseniospora*
**Basidiomycota**	**Tremellomycetes**	**incertae sedis**	*Tremellales*
			Cryptococcus
			Itersonilia
			Moniliella
	**Cystobasidiomycetes**	**Erythrobasidiaceae**	*Sporobolomyces*
			Rhodotorula
	**Microbotryomycetes**	**incertae sedis**	*Rhodosporidium*
			Sporobolomyces
	**Agaricomycetes**	**Coriolaceae**	*Formitopsis*
			Rigidosporus
		**Suillaceae**	*Suillus*
		**Russulaceae**	*Lactarius*
			Russula
		**Agaricaceae**	*Lepiota*
		**Boletaceae**	*Boletus*
			Xerocomus
		**Corticiaceae**	*Phanerochaete*
			Limonomyces
			Hyphodontia
		**Lachnocladiaceae**	*Vararia*
		**Crepidotaceae**	*Crepidotus*
		**Phanaerochaetaceae**	*Ceriporia*
		**Polyporaceae**	*Trichaptum*
		**Hymenochaetaceae**	*Fuscoporia*
		**Tricholomataceae**	*Paralepista*
		**Lycoperdaceae**	
		**Psathyrellaceae**	*Coprinopsis*
		**Trechisporaceae**	*Trechispora*
		**Sebacinaceae**	*Sebacina*
	**Exobasidiomycetes**	**Malasseziaceae**	*Malassezia*
	**Ustiloginomycetes**	**Glomosporiaceae**	*Thecaphora*
	**Wallemiomycetes**	**Wallemiaceae**	*Wallemia*
**Zygomycota**	**Zigomycetes**	**Mucoraceae**	*Mucor*
		**Lichtheimiaceae**	*Rhizomucor*
		**Rhizopodaceae**	*Rhizopus*
**Chytriodiomycota**	**Chytriodiomycetes**	**Powellomycetaceae**	*Powellomyces*
**Neocallimastigomycota**	**Neocallimastigomycetes**		

Supplementary Table 3| Association of Eurotiomycetes presence with altered metabolic parameters.

Women	Obese (Eurotiomycetes<1%)	Obese (Eurotiomycetes>1%)	Non-obese	*P*-value
**N**	7	14	12	
**Age**	48.00±8.52	46.21±7.26	43.08±9.11	0.4207
**BMI (kg/cm^2)**	46.00±5.10*	44.00±5.88*	22.67±3.34	1.70E-12
**SBP**	148.57±4.99*	134.28±19.20	121.83±15.75	0.005
**DBP**	8057±11.64*	71.78±9.06	64.83±10.24	0.009
**Fasting glucose (mg/dl)**	98.28±22.52	93.14±10.27	91.66±9.30	0.5759
**Glycated haemoglobin (%)**	5.82±0.57	5.82±0.34*	5.44±0.22	0.0244
**Fasting insulin (mU/l)**	16.8±11.9*#	7.06±3.62	3.90±3.39	0.0006
**HOMA (log)**	0.50±0.37*	0.13±0.27	-0.16±0.33	0.0005
**Cholesterol (mg/dl)**	220.71±38.53#	181.07±23.18	200.83±37.07	0.0364
**LDL Cholesterol (mg/dl)**	147±30.01	113.85±23.36	115.5±33.33	0.0423
**HDL Cholesterol (mg/dl)**	50.28±9.97*	50.78±7.96*	72.83±16.68	0.0001
**Fasting triglycerides (mg/dl)**	117.28±23.01*#	82.35±27.11	61.5±26.82	0.0004

BMI: Body mass index, SBP: Systolic Blood Pressure, DBP: Diastolic Blood Pressure, HOMA: homeostatic model assessment, LDL-cholesterol: low-density lipoprotein, HDL-cholesterol: high-density lipoprotein. Data are given as mean±S.D. using ANOVA test, Post hoc Bonferroni (normal distribution). *p<0.05 with respect to non-obese, #p<0.05with respect to obese with Eurotiomycetes>1%.

Suplemmentary Table 4| Eurotiomycetes abundance in mycobiota is associated with significant differences in plasma metabolites.

Potential Identity[1]	p.value	-Log10(p)	False discovery rate
**495.5044@13.79411**	3.28E-12	11.483	4.75E-09
**C23 H47 N3 O6 S**	5.16E-11	10.287	3.73E-08
**C28 H43 N**	1.74E-10	9.7601	8.37E-08
**C7 H12 N4 O**	1.41E-06	5.8512	0.00050891
**N-Acetyl-L-glutamic acid**	0.0061446	2.2115	0.86052
**C16 H30 N6 O2**	0.0065183	2.1859	0.86052
**C17 Cl N O4 S4**	0.0079103	2.1018	0.86052
**Hexadecanedioic acid**	0.0085971	2.0656	0.86052
**383.741@7.8528504**	0.011593	1.9358	0.86052
**481.3668@12.420571**	0.012404	1.9065	0.86052
**674.9938@0.3250769**	0.017577	1.7551	0.86052
**C12 H22 O2**	0.019729	1.7049	0.86052
**C31 H43 N3 O7**	0.019789	1.7036	0.86052
**679.0068@9.372867**	0.022009	1.6574	0.86052
**C8 H5 Cl N2 S3**	0.023505	1.6288	0.86052
**C6 H5 N3 O**	0.024037	1.6191	0.86052
**C9 H16 N2 S2**	0.024046	1.619	0.86052
**C24 H46 N6 O2 S2**	0.024143	1.6172	0.86052
**C30 H63 N9 S**	0.024146	1.6172	0.86052
**C31 H45 N O3**	0.024161	1.6169	0.86052
**762.0024@0.327303**	0.024232	1.6156	0.86052
**C19 H38 N8 O5**	0.024404	1.6125	0.86052
**C16 H30 O3**	0.024434	1.612	0.86052
**C16 H13 N O6**	0.024484	1.6111	0.86052
**C17 H40 N6 O3**	0.024547	1.61	0.86052
**C33 H67 N3 O**	0.024933	1.6032	0.86052
**630.2091@10.300446**	0.024949	1.6029	0.86052
**C10 H3 Cl O8**	0.024964	1.6027	0.86052
**C19 H26 N8**	0.025582	1.5921	0.86052
**C34 H67 N5 O4**	0.025852	1.5875	0.86052
**C22 H42 O4**	0.026323	1.5797	0.86052
**C17 H24 O3**	0.028789	1.5408	0.86052
**C23 H47 N7 O5**	0.028958	1.5382	0.86052
**C18 H35 N3 O2**	0.029824	1.5254	0.86052
**C17 H35 N7 O2**	0.03045	1.5164	0.86052
**C14 H27 N5 O3**	0.030601	1.5143	0.86052
**C20 H41 N13 O S**	0.030867	1.5105	0.86052
**C30 H42 Cl N3 O6**	0.031508	1.5016	0.86052
**C13 H15 Cl3 N12 O4 S3**	0.032627	1.4864	0.86052
**Paraxanthine**	0.03265	1.4861	0.86052
**785.6231@12.423894**	0.033071	1.4806	0.86052
**176.0804@0.61689997**	0.033798	1.4711	0.86052
**279.257@10.082667**	0.033798	1.4711	0.86052
**314.2859@0.48755556**	0.033798	1.4711	0.86052
**497.009@10.832399**	0.033816	1.4709	0.86052
**309.1215@1.0106999**	0.033844	1.4705	0.86052
**C5 H Cl2 N3**	0.033859	1.4703	0.86052
**453.1414@6.0391426**	0.033961	1.469	0.86052
**C38 H66 N4 O2**	0.034498	1.4622	0.86052
**281.2725@11.544888**	0.036132	1.4421	0.86052
**C29 H30**	0.037373	1.4274	0.86052
**C26 H41 N13 S**	0.038212	1.4178	0.86052
**C18 H45 N13 O5**	0.038475	1.4148	0.86052
**C16 H37 N3 O6**	0.038958	1.4094	0.86052
**Inosine 5'-monophosphate (IMP)**	0.04065	1.3909	0.86052
**C19 H32 N4 S**	0.041311	1.3839	0.86052
**1162.007@0.32976916**	0.042576	1.3708	0.86052
**C35 H33 Cl3 N4 O4**	0.04389	1.3576	0.86052
**C22 H50 N2 O8**	0.045353	1.3434	0.86052
**C24 H17 Cl N6 O14 S2**	0.046531	1.3323	0.86052
**2_3-Dihydroxy-pyridine**	0.04717	1.3263	0.86052
**C13 H16 N2 O4**	0.049647	1.3041	0.86052
**405.7564@7.911256**	0.049782	1.3029	0.86052

[1] Indicates potential identity of plasma metabolites significantly different by Student's t test in the two groups of patients differing in Eutiomycetes abundance. Metabolite identity are proposed on the basis of similarity of retention time, isotope distribution and exact mass with those present on the AMRT Database (Agilent). Alternatively, whenever a formula is present it has been calculated from isotope distribution, and otherwise metabolites are characterized by exact mass followed by retention time (in minutes); i.e. 405.7564@7.9112 describes the metabolite with a mass of 405.7564 appearing at 7.91 minutes.

Supplementary Figure 2 |

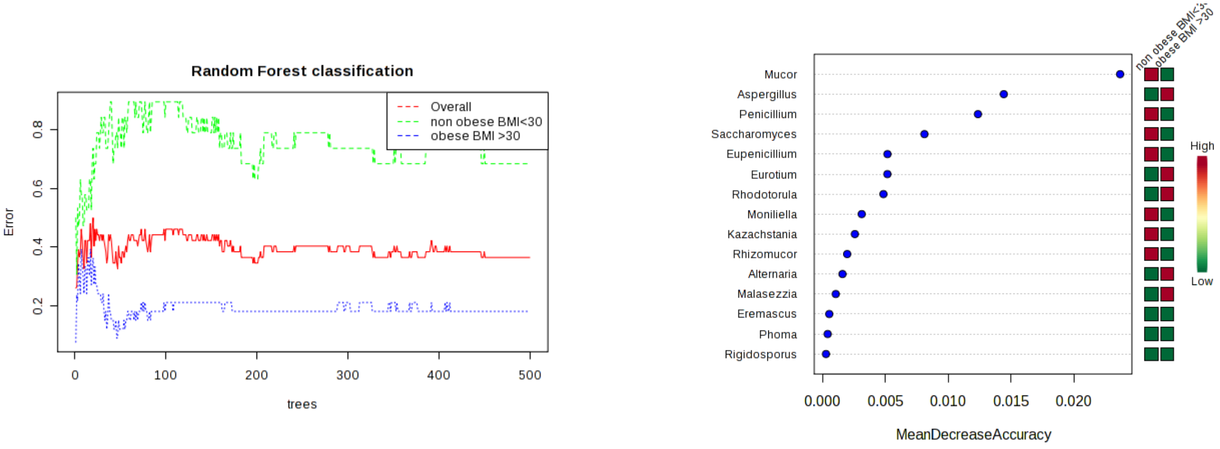

Error rate using different genus according random-forest classification (left), and genus contributing to the major differences in this system, as shown by mean decrease accuracy values (right).

Supplementary Figure 3 |

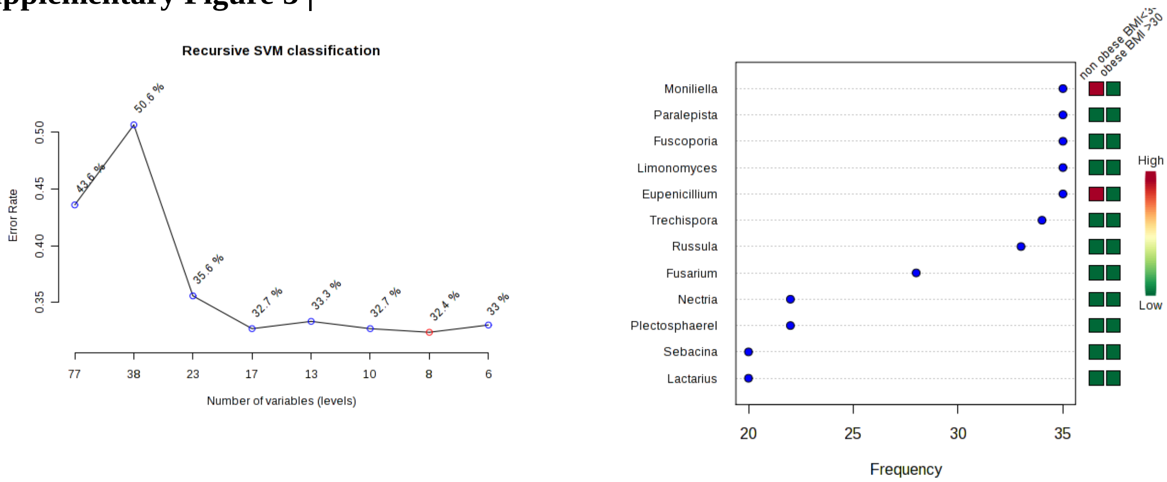

Error rate using different genus according recursive supported vector machine classification (left), and genus contributing to the major differences in this system (right).
